# The role of extracellular vesicles in the pathogenesis of gynecological cancer

**DOI:** 10.3389/fonc.2024.1477610

**Published:** 2024-09-26

**Authors:** Madhura Chatterjee, Saurabh Gupta, Tanmoy Mukherjee, Deepak Parashar, Umesh Kumar, Arindam Maitra, Kaushik Das

**Affiliations:** ^1^ Department of Biotechnology, Biotechnology Research and Innovation Council-National Institute of Biomedical Genomics, Kalyani, West Bengal, India; ^2^ Department of Biotechnology, Ganesh Lal Agarwal (GLA) University, Mathura, India; ^3^ Department of Cellular and Molecular Biology, The University of Texas at Tyler Health Science Center, Tyler, TX, United States; ^4^ Division of Hematology & Oncology, Department of Medicine, Medical College of Wisconsin, Milwaukee, WI, United States; ^5^ Department of Biosciences, Institute of Management Studies (IMS) Ghaziabad (University Courses Campus), Ghaziabad, Uttar Pradesh, India

**Keywords:** gynecological cancer, extracellular vesicles, biomarkers, therapeutic potential, cancer progression

## Abstract

Gynecological cancer, the most common form of cancers in women worldwide, initiates in the reproductive organs of females. More often, the common treatment measures, i.e. surgery, radiation, and medical oncology are found to be unsuccessful in the treatment of gynecological tumors. Emerging evidence indicates that extracellular vesicles (EVs) play a significant role in the pathogenesis of gynecological cancers by distinct mechanisms. The present review highlights how EVs contribute to the progression of different types of gynecological cancers such as cervical cancer, endometrial cancer, ovarian cancer, vaginal cancer, uterine sarcoma, gestational trophoblastic disease (GTD), and vulvar cancer. The primary focus is to understand how EVs’ cargo alters the phenotypic response of the recipient cells, thereby contributing to the progression of the disease, thus can be considered as a prognostic and diagnostic biomarker. A brief discussion on the role of EVs in the diagnosis and prognosis of different gynecological cancer types is also highlighted. Targeting the biogenesis of the EVs, their inside cargo, and EVs uptake by the recipient cells could be a potential therapeutic approach in the treatment of gynecological cancer beside conventional therapeutic means.

## Introduction

Gynecological cancers are defined as cancers which begin in the reproductive organs of females such as cervix, endometrium, fallopian tubes, ovaries, uterus, and vagina (1). It is considered as the commonest form of cancers in women worldwide which impose significant public health issues (2). In developing countries like India, gynecological cancers account for ~25% of all cancer types diagnosed among women aged up to mid-sixties (3). In India, cervical cancer ranks second in terms of both incidence and mortality (4). The present review begins with a brief introduction of different types of gynecological cancers including their mortality rate worldwide. The main section of the review focuses on understanding how extracellular vesicles (EVs) play their part in the progression of gynecological cancers by different mechanisms. The final part of the review highlights the role of EVs as biomarkers in different types of gynecological cancers.

## Types of gynecological cancers

**Figure 1:**
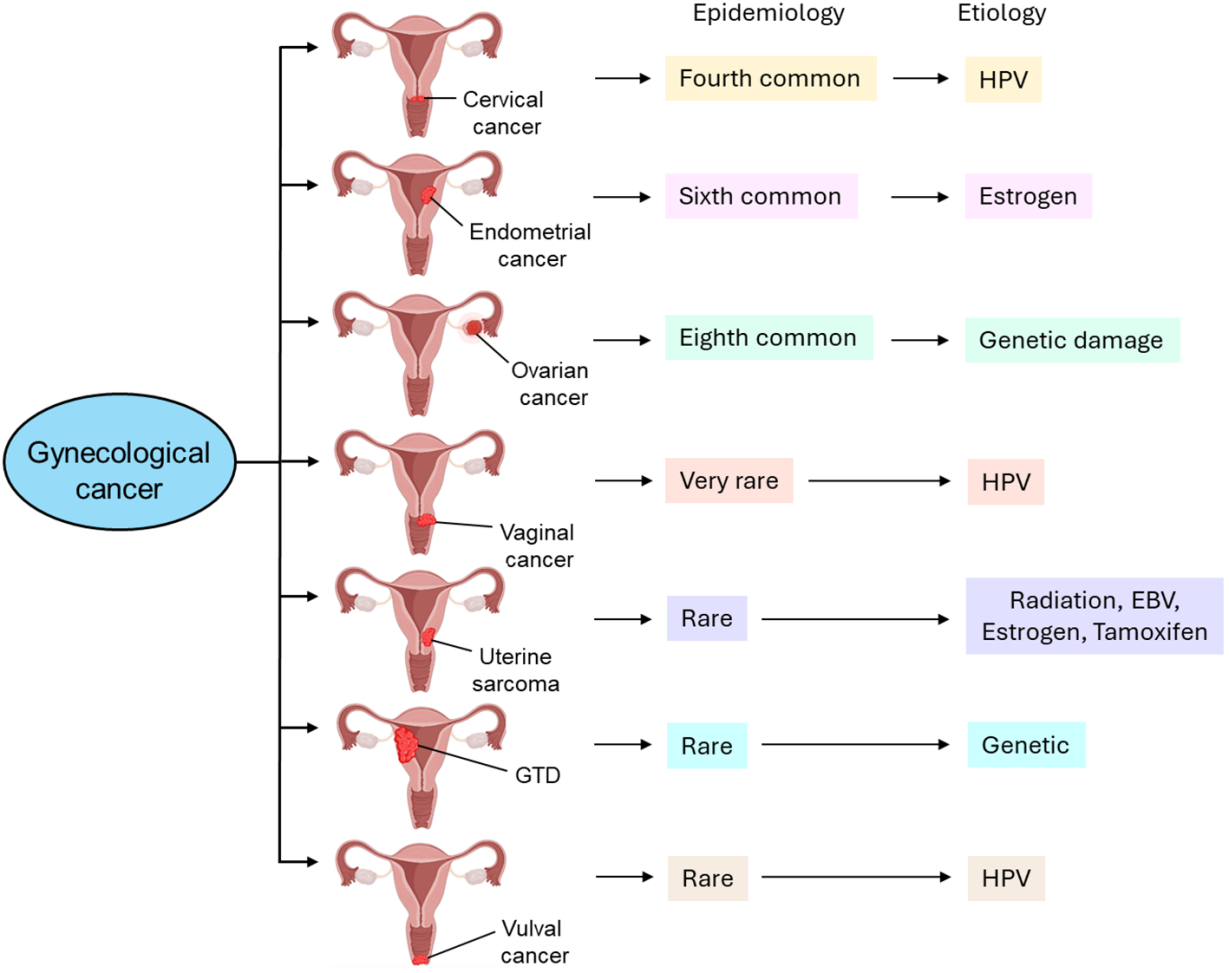
Different types of gynecological cancer, their epidemiology, and etiology. Cervical cancer is the fourth most common type of gynecological cancer which is caused by HPV infection. Endometrial cancer, the sixth most common type of gynecological cancer, is caused by estrogen. Ovarian cancer, the eighth most common type of gynecological cancer is caused by genetic damage. Vaginal cancer, which is very rare, is also caused by HPV infection. Uterine sarcoma is also rare and caused by radiation, EBV infection, estrogen, and tamoxifen. GTD and vulval cancer are also rare types of gynecological cancer whose etiology includes genetic and HPV, respectively. GTD, gestational trophoblastic disease; HPV, human papilloma virus; EBV, Epstein-Barr virus.

Gynecological tumors can be categorized into cervical cancer, endometrial cancer, ovarian cancer, vaginal cancer, uterine sarcoma, gestational trophoblastic disease (GTD), and vulvar cancer. **Table 1** briefly highlights the abundance, etiology, and cellular transformation mechanisms of different types of gynecological cancer. **Figure 1** also summarizes different forms of gynecological cancers, their epidemiology, and etiology.

**Table 1:** Different types of gynecological cancer; their rank according to the abundance, etiology, and cellular transformation mechanisms.

Gynecological cancer type	Rank	Etiology	Transformation mechanism	Reference/s
Cervical cancer	4	HPV	Viral E6 and E7 expression inactivates p53 and Rb of the host, promoting cellular transformation	(5)
			Down-regulation of FHIT expression promotes p16 and c-myc over-expression, triggering cervical cancer progression	(6)
			K-ras and H-ras mutations induce cervical cancer pathogenesis	(1)
			RCAS1 overexpression is associated with cervical cancer invasiveness	(7)
Endometrial cancer	6	Estrogen	Loss of PTEN expression is associated with type I endometrial cancer progression	(8)
			Mutations in K-ras and β-catenin also leads to type I endometrial cancer progression	(9)
			Microsatellite instability results intype I endometrial cancer	(10)
			Mutations in TP53 are associated with type II endometrial cancer	(11)
			HER2 overexpression, p16 inactivation, and E-Cadherin down- regulation is also observed in a few type II endometrial cancers	(12, 13)
Ovarian cancer	8	Genetic damage	K-ras and BRAF mutations in the development of ovarian cancer	(14)
			TP53 mutation and HER2, AKT2, and myc overexpression is associated with ovarian cancer development	(15, 16)
			~10% of ovarian cancer possesses mutations in BRCA1 and BRCA2 genes	(17)
			HB-EGF promotes proliferation and metastasis of ovarian cancer	(18)
			HB-EGF promotes proliferation and metastasis of ovarian cancer	(18)
Vaginal cancer	Rare	HPV	HPV infections lead to the development of vaginal cancer in younger women	(19)
			The expression of DDX48, erb-B3 binding protein, and biliverdin reductase is altered in vaginal carcinoma, which may play a role in vaginal carcinogenesis	(20)
Uterine sarcoma	Rare	Radiation	RTK and HER2 have been shown to play a key role in carcinosarcoma pathogenesis	(21)
		EBV	Rb-cyclin D plays an important role in leiomyosarcoma pathogenesis	(22)
		Radiation, estrogen, tamoxifen	Wnt pathway and histone de/ acetylation plays a key role in ESS pathogenesis	(23, 24)
GTD	Rare	Genetic	NALP7 mutation is shown to be responsible for GTD	(25)
Vulval cancer	Rare	HPV	EGFR and p53 overexpression is associated with poor prognosis of vulval cancer	(26)
			p73 overexpression is also observed in certain types of vulval cancer	(27)

HPV, human papilloma virus; Rb, retinoblastoma; FHIT, fragile histidine triad; ras, rat sarcoma; RCAS1, receptor binding cancer antigen expressed on SiSo cells; PTEN, phosphatase and TENsin homolog; TP53, tumor protein p53; HER2, human epidermal growth factor receptor 2; BRAF, rapidly accelerated fibrosarcoma homolog B; BRCA, breast cancer; HB-EGF, heparin-binding EGF-like growth factor; DDX48, DEAD (Asp-Glu-Ala-Asp) box protein 48; erb-B3, erythroblastic oncogene B; RTK, receptor tyrosine kinase; EBV, Epstein-Barr virus; Wnt, wingless/integrated; ESS, endometrial stromal sarcoma; GTD, gestational trophoblastic disease; EGFR, extracellular growth factor receptor.

### Cervical cancer

Cervical cancer, the malignant neoplasm, is originated from the cells in the uterine cervix which is further invaded into the female reproductive system (28). According to the World Health Organization (WHO), it is the fourth common cancer in women worldwide. The major causative agent for cervical cancer is human papilloma virus (HPV) (29). The 7,800 nucleotides long HPV DNA includes two open reading frames (ORFs), early- and late ORFs (30). Early ORFs codes for 7 proteins, named E1-7,which essentially control viral replication and host cell transformation (31). On the other hand, the late ORFs encoding proteins, L1 and L2 are the structural components of the virion (5). The integration of HPV DNA into the host genome leads to the expression of E6 and E7 which interact with host’ p53 and Rb, leading to their inactivation which ultimately results in cellular transformation (5). Therefore, the affinity of E6 and E7 to the host molecules determines the oncogenic potential of HPV, contributing to the pathogenesis of cervical cancer (32). However, emerging evidence indicates that the loss of chromosome 2q, -3p, and -11q as well as the addition of chromosome 1q and -3q are associated with the progression of cervical cancer (33). The fragile histidine triad gene (FHIT), located on chromosome 3p, is shown to be under-expressed in cervical cancer (34) which is accompanied by p16 and c-myc overexpression, thereby contributing to early progression of cervical cancer (6). Similarly, late events of cervical carcinogenesis are associated with mutations in K-ras and H-ras genes (1). On the other hand, a higher expression of RCAS1 is observed in invasive cervical carcinomas (7) which shows a positive correlation with cervical cancer progression.

### Endometrial cancer

Endometrial cancer is a tumor which arises in the inner epithelial lining of the uterus (35). It is the sixth common cancer developed among women worldwide. Endometrial cancer is further classified as (1) estrogen-related or type I or endometrioid carcinoma and (2) non-estrogen-related or type II or non-endometrioid carcinoma (36). Uncontrolled exposure of estrogen to preneoplastic lesion hyperplasia forms such type I endometrial cancer (37). Mechanistically, loss of PTEN expression (8) and mutations in K-ras and β-catenin genes (9) are shown to be associated with the progression of type I endometrial cancer. Additionally, microsatellite instability (MSI) also results in type I endometrial cancer (10). In contrast to type I, type II endometrial carcinoma is developed from atrophic endometrium (36). In majority of cases, mutations in TP53 gene is associated with type II endometrial cancer (11); whereas overexpression of HER2, inactivation of p16, and down-regulation of E-Cadherin expression are also observed in a few type II endometrial cancers (12, 13).

### Ovarian cancer

Ovarian cancer is defined as the malignancy of cells in the ovary (38). At present, it is the eighth most common cancer developed among women worldwide. The etiology of ovarian cancer includes damage to the genetic material (39). Mechanistically, mutations in K-ras and BRAF are shown to be associated with the development of ovarian cancer (14). In addition to these, mutations in TP53 and overexpression of AKT2, HER2 and myc also leads to ovarian cancer development (15, 16). However, ~10% of ovarian cancer has been reported to possess mutations in the genes, BRCA1 and BRCA2 (17), located in chromosomes 17q and 13q, respectively (40). Moreover, heparin-binding EGF (HB-EGF) plays a crucial role in the proliferation and metastasis of ovarian cancer (18), and HB-EGF inhibitors such as CRM197 may be used as a potential chemotherapeutic agent in the treatment of ovarian cancer (41).

### Vaginal cancer

Vaginal cancer is defined as cancer of the vagina without the evidence of vulval or cervical cancer or their presence in the past five years (42). Unlike the other types of gynecological cancers, vaginal cancer is very rare. Due to its rarity, the etiology of vaginal cancer is not completely understood. However, emerging evidence indicate that HPV infections may lead to the development of vaginal cancer in younger women (19) although in majority of instances, vaginal cancer is observed in older postmenopausal women (19). The expression of three signature proteins, DDX48, erb-B3 binding protein, and biliverdin reductase is shown to be significantly altered in vaginal carcinomas which are believed to play a major role in the pathogenesis of vaginal carcinoma (20).

### Uterine sarcoma

Uterine sarcomas are originated from the smooth muscles and connective tissues of the uterus (43). It accounts for ~1% of all gynecological cancers, therefore is also considered as a rare type of gynecological tumor (44). Uterine sarcomas have a few variants, carcinosarcoma, leiomyosarcoma, and endometrial stromal sarcoma (ESS) (45). The etiology of uterine sarcoma appears to be epigenetic. For example, radiation is the probable cause of carcinosarcoma (46). Receptor tyrosine kinase (RTK) and HER2 are shown to play a key role in carcinosarcoma pathogenesis and inhibitors against RTK and HER2 are found to be quite effective against carcinosarcoma (21). Epstein-Barr virus (EBV) infection has been shown to be associated with leiomyosarcoma (47). Mechanistically, ~90% of leiomyosarcoma cases have defects in the Rb-cyclin D pathway, which demonstrates the crucial role Rb-cyclin D pathway in the pathogenesis of leiomyosarcoma (22). Radiation and prolonged use of estrogen or tamoxifen have been shown to be the etiology for ESS (48). Mechanistically, the deregulation of Wnt signaling pathway is responsible for ESS pathogenesis (23). In another study, histone de/acetylation is shown to play a crucial role in the progression of ESS (24) and HDAC inhibitors could be used as potential therapeutics against ESS (23).

### GTD

GTD is a rare form of gynecological cancer (49) with an incidence of 1 to 2 per 1000 pregnancies (50), resulted from abnormal fertilization (51). The etiology of GTD is shown to be genetic aberration. In this case, fertilization of an ovum without maternal chromosomes with a sperm forms the complete hydatidiform mole which constitutes all paternal chromosomes (52). Genes responsible for GTD are shown to be located in chromosome 19q13.3–13.4 (53), in which NALP7 mutation is predominantly observed (25).

### Vulval cancer

Vulval cancer, the cancer of the vulva in postmenopausal women (54), is another type of rare gynecological cancer which accounts for 2-5% of all gynecological cancers (54). The most common subtype of vulval cancer is the squamous cell carcinoma (SCC) (54). HPV is the main causal agent of vulval cancer (55) although HPV-negative vulval cancer also exists (27). Overexpression of EGFR and p53 is shown to be associated with poor prognosis of vulval cancer (26). In addition, p73 overexpression is also observed in certain types of vulval cancer (27).

### The role of EVs in gynecological cancer

#### EVs: a general overview

**Figure 2:**
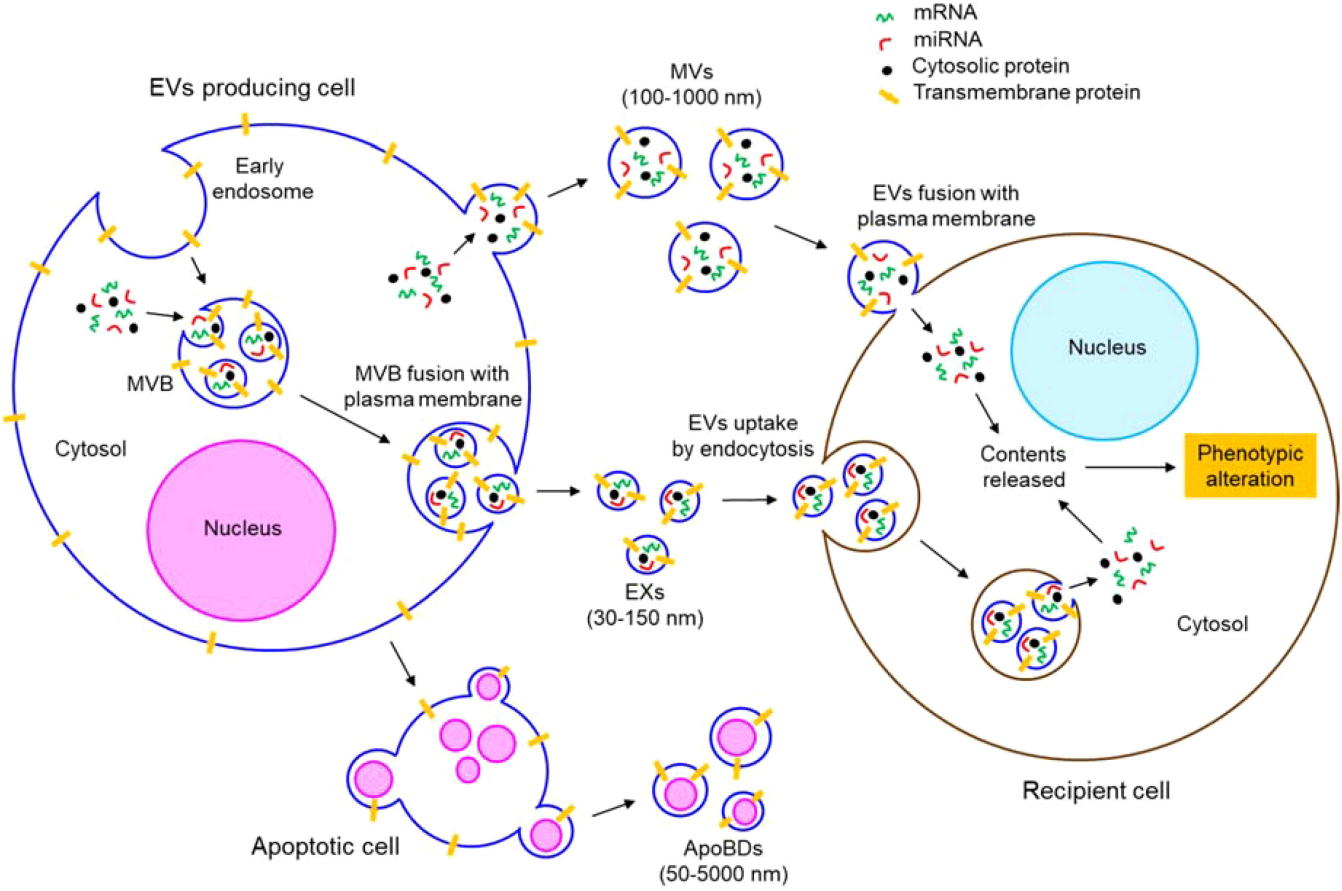
Biogenesis and uptake of different types of EVs. EVs comprise of MVs, EXs, and ApoBDs. MVs, 100-1000 nm in size, are produced by plasma membrane outward budding. EXs are 30-150 nm in size and of endocytic origin. Invagination of plasma membrane forms early endosomes. Invagination of early endosomal membrane generates EXs which mature into MVB. MVB fuses with the plasma membrane to release the EXs outside the cells. ApoBDs are generated from apoptotic cells having varying size (50-5000 nm). EVs are taken up by the recipient cells either by direct fusion with the plasma membrane or by endocytosis. In both the mechanisms, eventually, the contents (such as mRNAs, miRNAs, proteins, etc.) of the EVs are released into the cytosol of the recipient cells, thereby altering the phenotypes of the EVs fused recipient cells.

EVs are lipid bilayer enclosed, nano-sized particles which are released from almost every cell type into the extracellular environment (56). EVs represent a third mechanism of cell-to-cell communication beside the direct cell-to-cell contact and cellular secretary molecules (57). EVs transfer biomolecules such as DNA, RNA, microRNA (miRNA), long non-coding RNA (lncRNA), circular RNA, protein, lipid, metabolite etc. between the cells, thereby altering the phenotypes of the target recipient cells (58–65). EVs are abundantly found in biological fluids like blood, urine, saliva, breast milk, cerebrospinal fluid etc. and even in the interstitial spaces between the cells (66–70). EVs are readily taken up by the recipient cells either by direct fusion of EVs’ membrane with the recipient cells’ plasma membrane or by endocytic mechanism (60, 71). EVs can be broadly classified according to the biogenetic mechanism, size distribution, and function into microvesicles (MVs), exosomes (EXs), and apoptotic bodies (ApoBDs). **Figure 2** briefly illustrates the biogenetic mechanism of different types of EVs and their fusion with target recipient cells.

MVs (also called microparticles; MPs or ectosomes) are produced by outward budding of the plasma membrane of a cell (61). Actomyosin reorganization is shown to play a crucial role in the biogenesis of MVs (61). Therefore, regardless of the originating cell, both cytosolic and membrane-associated proteins such as tetraspanins, integrins, cytoskeletal proteins, heat shock proteins, and proteins associated with post-translational modifications are often found abundantly in the MVs (69). MVs range in size from 100 nm to 1µm (70).

Unlike MVs, EXs have a diameter range of 30-150 nm (72). EXs are of endocytic origin (69); first the invagination of the plasma membrane forms large endosomes which are further invaginated to produce smaller EXs within the endosomes that are matured into multivesicular bodies (MVBs) (69). MVBs eventually fuse with the plasma membrane to release the EXs outside (69). Therefore, the endosomal sorting complexes required for the transport (ESCRT) pathway-associated proteins such as Alix, TSG101 etc. are abundantly found in the EXs (73, 74).

The other type of EVs, ApoBDs, having a broader diameter range between 50 nm to 5 µm, are generated from apoptotic cells (75). Apoptotic stimuli-induced cellular contraction generates a significant hydrostatic pressure which segregates the plasma membrane from the cytoskeleton, leading to the release of ApoBDs (76). Different cell organelle markers such as GRP78 for Golgi and endoplasmic reticulum, HSP60 for mitochondria, histones for nucleus are abundantly found in the ApoBDs (76).

#### EVs in gynecological cancer

Emerging evidence indicates that EVs play a crucial role in the development and progression of gynecological cancers. The present section briefly highlights how EVs contribute to the progression of different types of gynecological cancers. **Table 2** summarizes EVs’ role in various gynecological tumors.

**Table 2:** The role of EVs in the progression of different types of gynecological cancers.

Gynecological cancer type	EV component	Mechanism	Reference/s
Cervical cancer	Wnt7b mRNA	E6 oncoproteins of HPV 16/18 induce Wnt7b mRNA in cervical cancer cells, leading to the release Wnt7b mRNA positive EVs which promote endothelial proliferation and angiogenesis.	(77)
	Cytochrome P450 and HPV oncoproteins	HPV-infected cervical cancer cell-derived EVs induce replication of HIV-1 in macrophages via the transfer of cytochrome P450 and HPV oncoproteins	(78)
	MCM3AP-AS1	Cervical cancer cell-derived EVs transfer MCM3AP-AS1 to endothelial cells, thereby inducing angiogenesis through binding miR-93 and inducing miR-93 target, p21 expression, further facilitating tumor growth	(79)
	miR-144-3p	hBMSC-EVs transfer miR-144-3p to cervical cancer cells and target CEP55, inhibiting proliferation, migration, and invasion of cancer cells while promoting invasion of cancer cells while promoting apoptosis	(80)
	miR-331-3p	hBMSC-EVs also transfer miR-331-3p to cervical cancer cells which targets DNMT3A and down-regulates the methylation of LIMS2 to inhibit the growth of cervical tumor	(81)
Endometrial cancer	TC0101441	TC0101441 is transferred from H-ECSCs to L-ECSCs via the EVs, promoting migration and/or invasion of the endometriosis	(82)
	LGALS3BP	Endometrial tumor-derived EVs are enriched with LGALS3BP with epithelial-like properties which facilitate secondary colonization of the tumor	(83)
	hsa_circ_0001610	M2-macrophage-derived EVs transfer hsa_circ_0001610 to the endometrial cells, thereby targeting miR-139-5p expression, leading to cyclin B1 expression and conferring tumor radio resistance	(84)
	miR-302a	hUCMSC-EVs are enriched with transfer hsa_circ_0001610 to the endometrial cells, thereby targeting miR-139-5p expression, leading to cyclin B1 expression and conferring tumor radio resistance	(84)
	miR-302a	hUCMSC-EVs are enriched with miR-302a which targets cyclin D and AKT pathway in endometrial cancer cells, thereby inhibiting tumor proliferation and migration	(85)
	miR-320a	miR320a over-expressed EVs from CAFs inhibit HIF-1α expression in endometrial cancer cells, thereby down-regulating VEGF-A expression and associated tumor proliferation	(86)
	carboplatin, paclitaxel	MSC-EVs, loaded with carboplatin and paclitaxel induce endometrial cancer cell apoptosis whereas inhibiting cell migration and invasion via down-regulating Rac1/NF-κB- dependent expression of MMP-2	(87)
Ovarian cancer	MMP-1 mRNA	Ovarian cancer cell-derived EVs transfer MMP-1 mRNA to mesothelial cells, leading to apoptosis, thereby facilitating peritoneal dissemination of metastatic ovarian cancer	(88)
		Cisplatin-treated ovarian cancer cells release EVs which promote invasion and cisplatin-resistance to recipient bystander cells via the activation of p38 and JNK signaling pathway	(89)
	SLPI	FAP^high^α-SMA^low^ subpopulation of CAFs release SLPI-positive EVs which promote the proliferation migration, invasion, and adhesion of ovarian cancer cells via the activation of PI3K/AKT pathway	(90)
	miR-18a-5p	hMSC-EVs transfer miR-18a-5p to ovarian cancer cells and inhibit their proliferation, migration, invasion, and chemoresistance via targeting NACC1	(91)
	miR-424	MSC-EVs transfer miR-424 to ovarian cancer cells, leading to the downregulation of proliferation, migration, and invasion via targeting MYB. miR-424 of the MSC-EVs also suppress angiogenesis by reducing the expression of VEGF and VEGFR	(92)
	FasL, TRAIL	Ascites of ovarian cancer patients bear FasL- and TRAIL-positive EVs which trigger immune evasion by inducing apoptosis of immune cells	(93)
	ARG1	ARG1 in ovarian cancer ascites-derived EVs suppresses peripheral T-cells, leading to immune evasion and promoting tumor growth	(94)
	circ-0001068	circ-0001068 level in the serum EVs of ovarian cancer patients are significantly upregulated which is transferred to T-cells, leading to T-cell exhaustion and tumor immune evasion	(95)
Vaginal cancer		EVs from *G. vaginalis* and *M. mulieris* promote TLR-2-specific inflammatory response to trigger adverse reproductive outcomes	(96)
	Unique proteins and metabolites	EVs from *Lactobacillus* spp. transfer unique proteins and metabolites to CD4^+^ T-cells, thereby preventing the attachment and entry of HIV-1 into the target cells	(97)
	TIMP-2, TGFβ, ABCC4	Vaginal fibroblast-derived EVs of SUI patients down-regulate the collagen content, proliferation, and migration of normal fibroblasts via the transfer of TIMP-2, TGFβ, and ABCC4	(98)
Uterine sarcoma	miR-369-3p, miR-654-3p	The expression of miR-369-3p and miR-654-3p is up-regulated in the EVs derived from cell lines, tissues, and sera of ULMS patients which converts normal fibroblasts into CAFs	(98)
Vulval cancer	UCA1	CAF-EVs transfer UCA1 to VSCC cells and confer cisplatin resistance through the regulation of miR-103a/ WEE1 axis	(99)

Wnt, wingless/integrated; HPV, human papilloma virus; EVs, extracellular vesicles; HIV-1, human immunodeficiency virus 1; MCM3AP-AS1, micro-chromosome maintenance protein 3-associated protein antisense RNA 1; hBMSC, human bone marrow mesenchymal stem cell; CEP55, centrosomal protein of 55 kDa; DNMT3A, DNA methyltransferase 3 alpha; LIMS2, LIM zinc finger domain containing 2; H-ECSC, TC0101441 high expressing endometriotic cyst stromal cell; L-ECSC, TC0101441 low expressing endometriotic cyst stromal cell; LGALS3BP, galectin-3-binding protein; hUCMSC, human umbilical cord mesenchymal stem cell; miR, microRNA; CAFs, cancer-associated fibroblasts; HIF-1α, hypoxia-inducible factor 1α; VEGF, vascular endothelial growth factor; hMSC, human mesenchymal stem cell; Rac1, Ras-related C3 botulinum toxin substrate 1; NF-κB, Nuclear factor kappa B; MMP, matrix metalloproteinase; JNK, Jun N-terminal kinase; FAP, fibroblast activation protein-α; α-SMA, α smooth muscle cell actin; SLPI, secretory leukocyte protease inhibitor; PI3K, phosphoinositide 3-kinase; NACC1, nucleus accumbens-associated protein 1; MYB, myeloblastosis viral oncogene homolog; VEGFR, VEGF receptor; TLR-2, Toll-like receptor 2; CD, cluster of differentiation; SUI, stress urinary incontinence; TIMP-2, tissue inhibitor of metalloproteinases 2; TGFβ, transforming growth factor-beta; ABCC4, ATP-binding cassette sub-family C member 4; ULMS, uterine leiomyosarcoma; UCA1, urothelial cancer-associated 1; VSCC, vulvar squamous cell carcinoma.

#### EVs in cervical cancer

**Figure 3:**
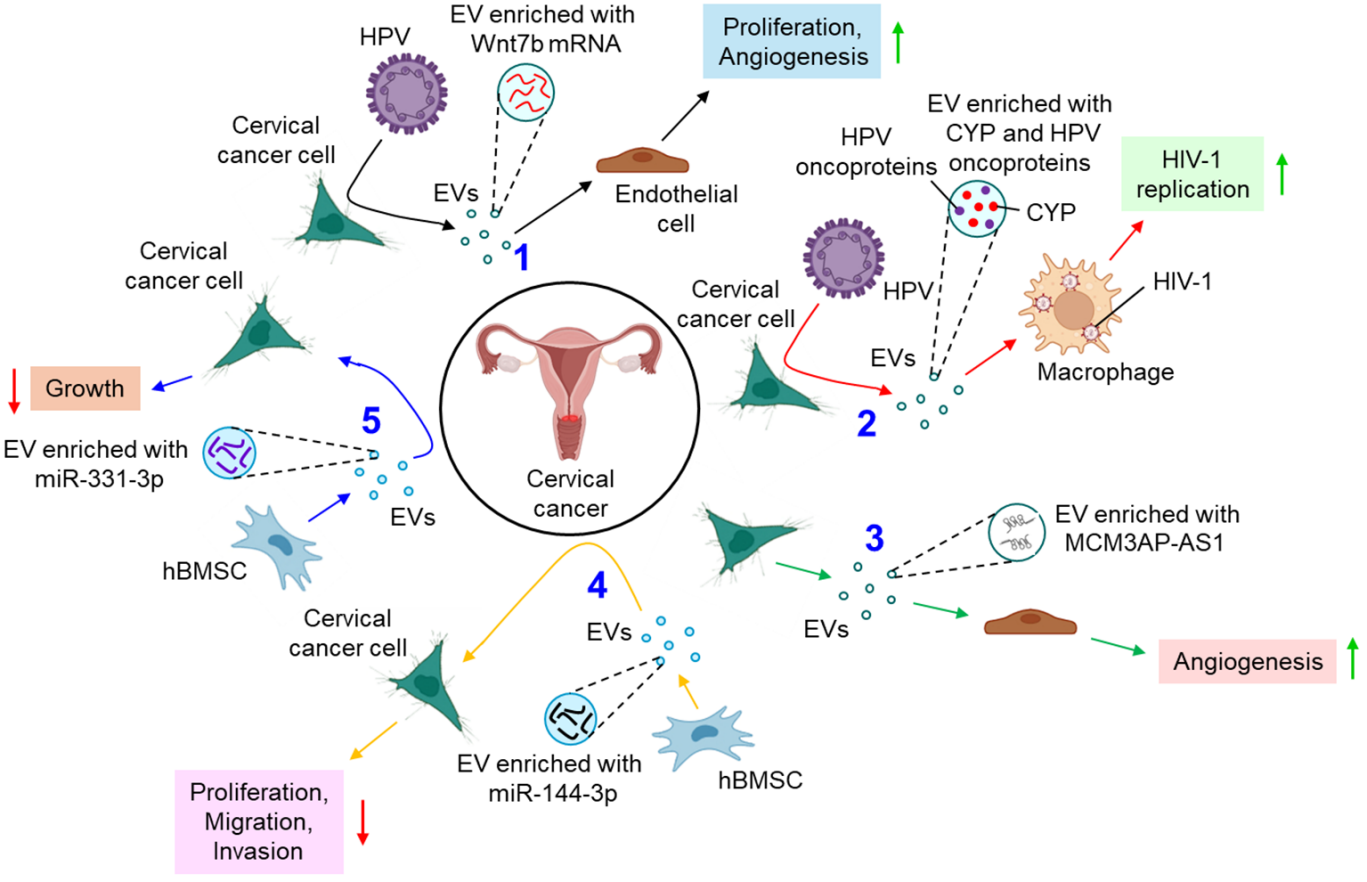
The role of EVs in cervical cancer. 1. HPV oncoproteins lead to the release of Wnt7b mRNA-enriched EVs from cervical cancer cells which promote proliferation and angiogenesis of endothelial cells. (black arrows) 2. HPV-infected cervical cancer cell-derived EVs promote replication of HIV-1 in macrophages (red arrows) 3. Cervical cancer cell-derived EVs transfer MCM3AP-AS1 to endothelial cells to promote angiogenesis (green arrows) 4. hBMSC-EVs transfer miR-144-3p to cervical cancer cells and down-regulate their proliferation, migration, and invasion. (yellow arrows) 5. hBMSC-EVs also release miR-331-3p-enriched EVs which inhibit the growth of cervical cancer cells. (blue arrows). Green upward arrows indicate up-regulation; Red downward arrows indicate down-regulation. HPV, human papilloma virus; EV, extracellular vesicle; CYP, cytochrome P450; HIV-1, human immunodeficiency virus 1; MCM3AP-AS1, micro-chromosome maintenance protein 3-associated protein antisense RNA 1; hBMSC, human bone marrow mesenchymal stem cell; miR, microRNA.

Several studies delineate the active participation of EVs in the progression of cervical cancer. For example, E6 oncoproteins of HPV 16/18 are shown to induce the expression of Wnt7b mRNA in cervical cancer cells, resulting in the release of Wnt7b mRNA-enriched EVs (77). These EVs transfer Wnt7b mRNA to the endothelial cells leading to proliferation and angiogenesis by influencing β-catenin signaling (77). HPV-infected cervical cancer cell-secreted EVs are also shown to increase the replication of human immunodeficiency virus (HIV)-1 in infected macrophages through the transfer of cytochrome P450 (CYP) and HPV oncoproteins (78). In another study, cervical cancer cell-derived EVs are appeared to carry a long non-coding RNA (lncRNA), micro-chromosome maintenance protein 3-associated protein antisense RNA 1 (MCM3AP-AS1) which is transferred through the EVs to the recipient endothelial cells, leading to enhanced angiogenesis, via binding miR-93 and up-regulating its target, p21 expression (79). This in turn facilitates tumor growth (79). A bunch of studies also indicate that EVs often impede the progression of cervical cancer by various mechanisms. Human bone marrow mesenchymal stem cell (hBMSC)-derived EVs carry miR-144-3p to cervical cancer cells and target centrosomal protein of 55 kDa (CEP55), leading to the suppression of cancer cell proliferation, migration, and invasion while promoting apoptosis, ultimately inhibiting the progression of cervical cancer (80). hBMSC-EVs are also shown to deliver miR-331-3p to the cervical cancer cells and target DNA methyltransferase 3 alpha (DNMT3A) to reduce the methylation of LIM zinc finger domain containing 2 (LIMS2), thereby perturbing the growth of cervical tumors (81). **Figure 3** briefly summarizes how EVs influence the progression of cervical cancer by different mechanisms.

#### EVs in endometrial cancer

**Figure 4:**
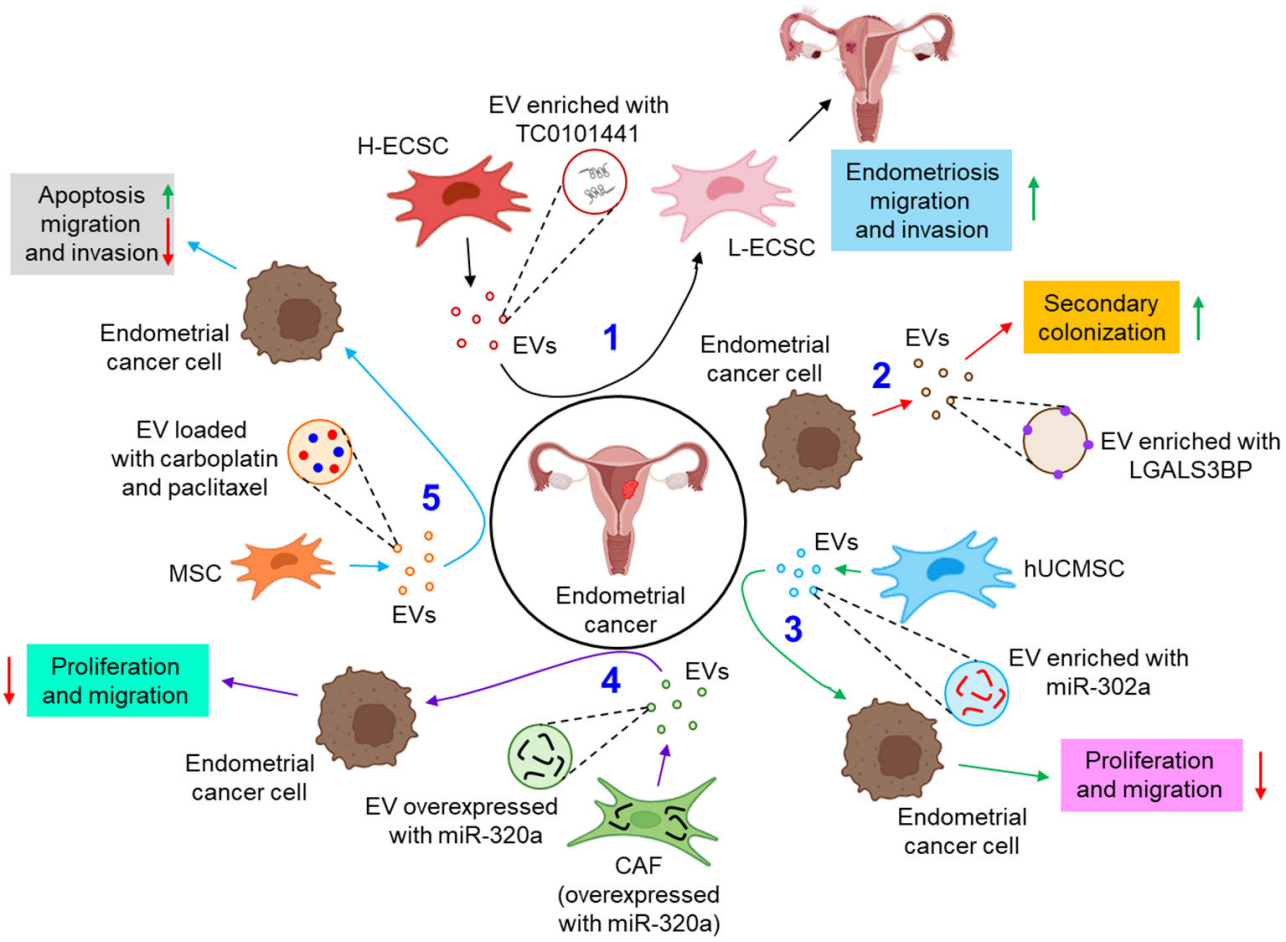
The role of EVs in endometrial cancer. 1. EV-mediated transfer of TC0101441 from H-ECSC to L-ECSC contributes to endometriosis migration and invasion. (black arrows) 2. Endometrial cancer cell-derived EVs are enriched with LGALS3BP which helps in secondary colonization of the tumor. (red arrows) 3. hUCMSC-EVs, enriched with miR-302a, inhibit the proliferation and invasion of the endometrial cancer cell. (green arrows) 4. CAF, overexpressed with miR-320a, tends to release miR-320a enriched EVs which down-regulate endometrial tumor cell proliferation and migration. (violet arrows) 5. Carboplatin and paclitaxel loaded MSC-EVs enhance apoptosis and inhibit migration and invasion of endometrial cancer cells. (sky arrows). Green upward arrows indicate up-regulation; Red downward arrows indicate down-regulation. H-ECSC, TC0101441 high expressing endometriotic cyst stromal cell; EVs, extracellular vesicles; L-ECSC, TC0101441 low expressing endometriotic cyst stromal cell; LGALS3BP, galectin-3-binding protein; hUCMSC, human umbilical cord mesenchymal stem cell; miR, microRNA; CAF, cancer-associated fibroblast; MSC, mesenchymal stem cell.

EVs are also associated with the pathogenesis of endometrial cancer. For example, a lncRNA, TC0101441 is shown to be transferred from TC0101441 high expressing endometriotic cyst stromal cells (H-ECSCs) to TC0101441 low expressing ECSCs (L-ECSCs) through the EVs, thereby promoting endometriosis migration and/or invasion (82). Moreover, circulating endometrial tumor cell-derived EVs are found to be enriched with the adhesion protein, galectin-3-binding protein (LGALS3BP) which imparts the epithelial-like properties of the EVs, facilitating the secondary colonization of the tumor (83). miRNAs are often considered as predictive and diagnostic biomarkers for endometriosis (100). Gu et al. further demonstrated that M2-macrophage-derived EVs transfer circular RNA, hsa_circ_0001610 to endometrial cancer cells, leading to a downregulation of miR-139-5p expression, thereby inducing the expression of miR-139-5p target, cyclin B1 and thus conferring tumor radio resistance (84). Li et al. have demonstrated that human umbilical cord mesenchymal stem cell (hUCMSC)-released EVs are enriched with miR-302a which targets cyclin D1 and AKT signaling pathway in endometrial cancer cells, thereby inhibiting tumor proliferation and migration (85). Thus, miR-302a loaded EVs can be used as potential therapeutics in the treatment of endometrial cancer. In another study, miR-320a over-expressed EVs from cancer associated fibroblasts (CAFs) are shown to down-regulate hypoxia-inducible factor 1α (HIF-1α) in endometrial cancer cells, leading to the inhibition of vascular endothelial growth factor A (VEGF-A) expression and associated tumor proliferation (86). In a recent study by Ma et al., mesenchymal stem cell (MSC)-derived EVs loaded with therapeutic drugs, carboplatin and paclitaxel have been shown to induce apoptosis while perturbing endometrial cancer cell migration and invasion via Rac1/NF-κB-mediated down-regulation of matrix metalloproteinase 2 (MMP-2) expression (87). **Figure 4** briefly illustrates how EVs influence the progression of endometrial cancer by different mechanisms.

#### EVs in ovarian cancer

**Figure 5:**
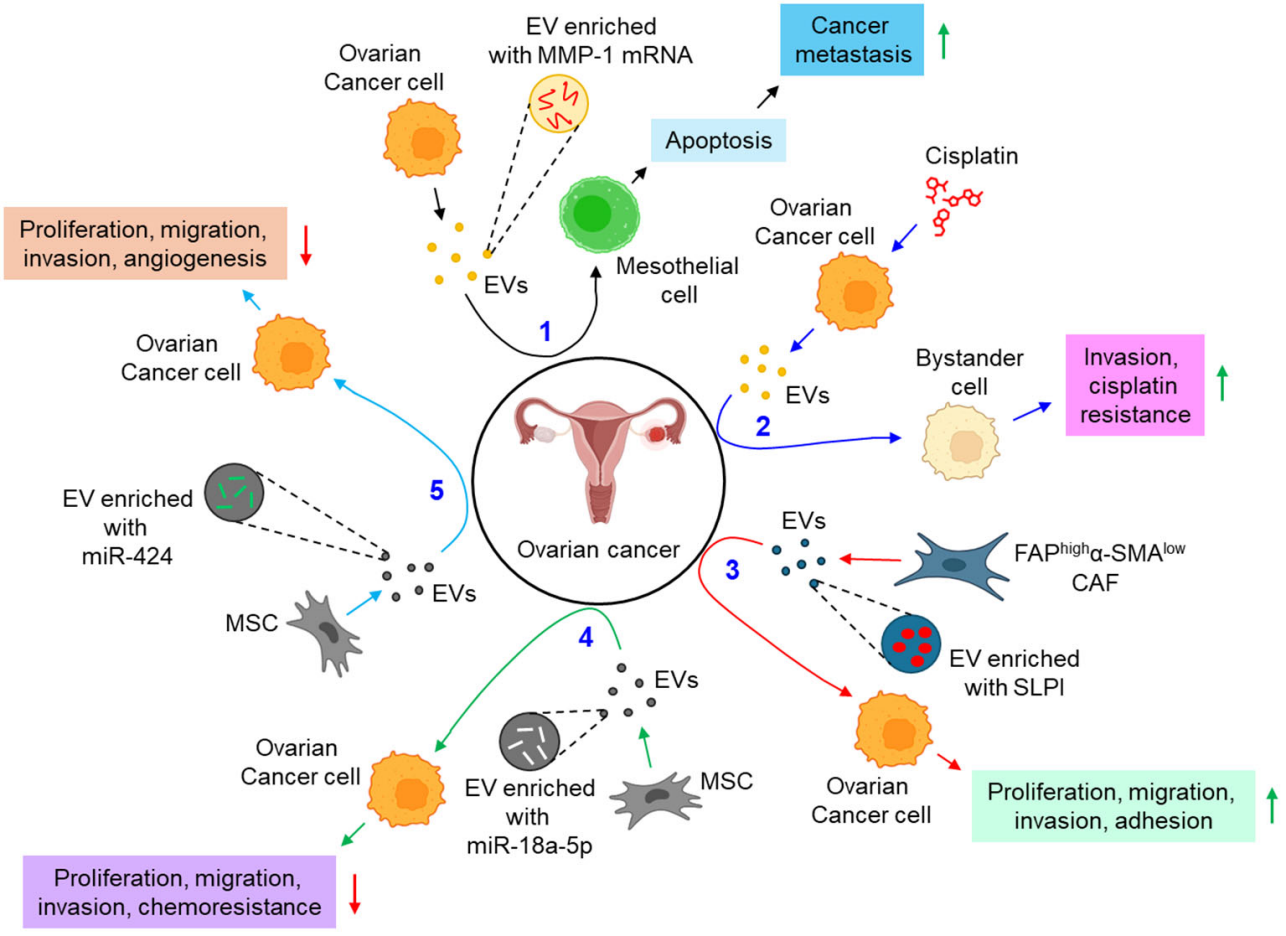
The role of EVs in ovarian cancer. 1. Ovarian cancer cell-derived EVs transfer MMP-1 mRNA to mesothelial cell, leading to mesothelial apoptosis, thereby augmenting cancer metastasis. (black arrows) 2. Cisplatin-treated ovarian cancer cell-derived EVs promote invasion and impart cisplatin resistance to bystander cells. (blue arrows) 3. FAP^high^α-SMA^low^ CAF-EVs promote proliferation, migration, invasion, and adhesion of ovarian cancer cells via the transfer of SLPI. (red arrows) 4. MSC-EVs transfer miR-18a-5p to ovarian cancer cells, thereby down-regulating cancer proliferation, migration, invasion, and chemoresistance. (green arrows) 5. MSC-EVs also transfer miR-424 to ovarian cancer cells, and hence perturbing cancer proliferation, migration, invasion, and angiogenesis. (sky arrows). Green upward arrows indicate up-regulation; Red downward arrows indicate down-regulation. EVs, extracellular vesicles; FAP, fibroblast activation protein-α; α-SMA, α smooth muscle cell actin; CAF, cancer-associated fibroblast; SLPI, secretory leukocyte protease inhibitor; MSC, mesenchymal stem cell; miR, microRNA.

A growing body of evidence identifies EVs to be a critical regulator in the development of metastasis and chemoresistance in epithelial ovarian cancer, mainly via augmenting epithelial to mesenchymal transition and tumor immune evasion (101). The study by Yokoi et al. indicates that EVs from ovarian cancer cells efficiently transfer MMP1 mRNA to mesothelial cells, leading to mesothelial apoptosis, which facilitates peritoneal dissemination of metastatic ovarian cancer (88). In a contemporary study, Samuel et al. have demonstrated that treatment of chemotherapeutic drug, cisplatin to ovarian cancer cells leads to the release of pro-cancerous EVs which not only induce invasion to the recipient bystander cells, but also confer cisplatin resistance through the activation of p38 and JNK signaling pathway (89). FAP^high^α-SMA^low^ subpopulation of CAFs are shown to release secretory leukocyte protease inhibitor (SLPI) through the EVs which facilitates the proliferation, migration, invasion, and adhesion of ovarian cancer cells via the activation of PI3K/AKT pathway (90). In contrast, human MSC (hMSC)-derived EVs inhibit the proliferation, migration, invasion, and chemotherapy resistance of ovarian cancer cells via the transfer of miR-18a-5p and targeting nucleus accumbens-associated protein 1 (NACC1) (91). Similarly, MSC-EVs are also shown to transfer miR-424 to ovarian cancer cells, thereby down-regulating their proliferation, migration, and invasion, probably by targeting myeloblastosis viral oncogene homolog (MYB) (92). The study also delineates that miR-424 transfer through MSC-EVs reduce the expression of endothelial VEGF and VEGFR, thereby suppressing tumor angiogenesis (92). Emerging evidence indicates that genital microbiome plays a key role in genital dysbiosis and development of cervical- and endometrial cancer (102). However, understanding microbiome’s role in ovarian cancer development requires further investigations with robust methodologies which will aid in the development of novel preventive and therapeutic drugs (102). In this context, how the microbiome profile influencing the EVs population, and their characteristics can open a new therapeutic window in the treatment of ovarian cancer. A growing body of evidence also indicates that EVs actively carry immunosuppressors which aid evading host immune response and promoting progression of ovarian cancer. For example, ascites of ovarian cancer patients carries FasL- and TRAIL-positive EVs which facilitate immune evasion by inducing apoptosis of immune cells (93). Similarly, ARG1 in the EVs of ovarian cancer patients’ ascites suppresses peripheral T-cells, leading to immune evasion, thereby facilitating tumor growth (94). Moreover, the expression of circular RNA, circ-0001068 is shown to be significantly elevated in the serum EVs of ovarian cancer patients which induces the expression of PD-1 in T-cells, leading to T-cell exhaustion and promotion of tumor growth (95). In addition to the above, EV metabolites often result in the metabolic reprogramming of the recipient cells. For example, CAF-EVs are shown to carry amino acids and TCA cycle intermediates which are readily taken up by prostate cancer cells, leading to tumor growth and metastasis (103). However, EV-mediated metabolic reprogramming of gynecological cancers including ovarian cancer remains ill-defined. The reasons include difficulties in identifying EV metabolites for effective phenotypic alterations, different cell culture conditions often result in the enrichment of different metabolites into the EVs, the genetic variant associated with different metabolites enrichment into the EVs remains unexplored, and difficulties in EV isolation and purification for metabolites characterization (104). **Figure 5** illustrates how EVs are associated with the progression of ovarian cancer.

#### EVs in vaginal cancer

**Figure 6:**
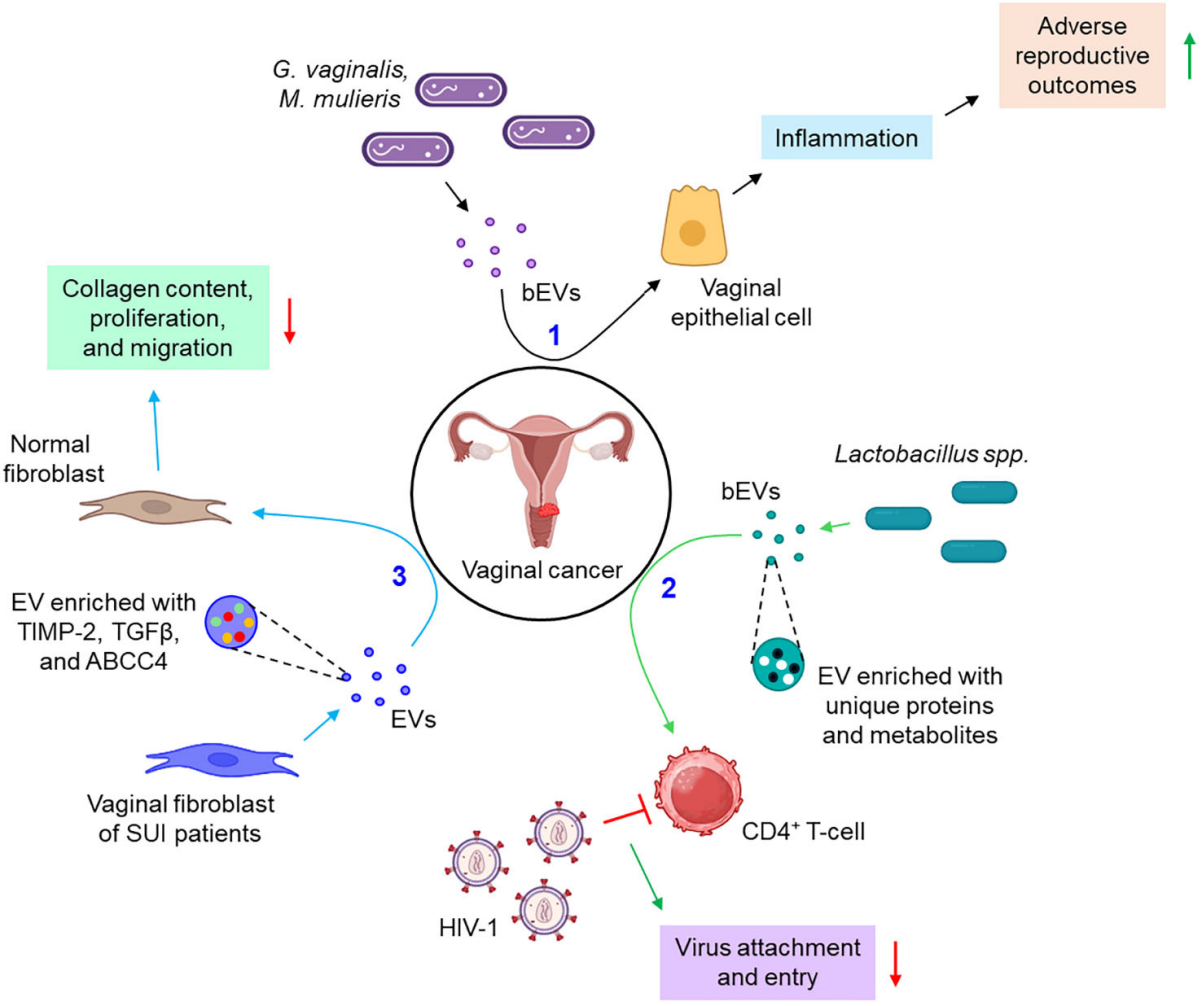
The role of EVs in vaginal cancer. 1. bEVs from *G. vaginalis* and *M. mulieris* induce inflammation of vaginal epithelial cells, thereby leading to adverse reproductive outcomes. (black arrows) 2. *Lactobacillus* spp.-derived bEVs are enriched with several unique proteins and metabolites which prevent the attachment and entry of HIV-1 to CD4^+^ T-cells. (green arrows) 3. Vaginal fibroblasts release TIMP-2, TGFβ, and ABCC4-enriched EVs which down-regulate the collagen content, proliferation, and migration of normal fibroblasts. (sky arrows). Green upward arrows indicate up-regulation; Red downward arrows indicate down-regulation. bEVs, bacterial extracellular vesicles; CD, cluster of differentiation; SUI, stress urinary incontinence; TIMP-2, tissue inhibitor of metalloproteinases 2; TGFβ, transforming growth factor-beta; ABCC4, ATP-binding cassette sub-family C member 4.

A few instances report the active participation of the EVs in the pathogenesis of vaginal cancer. Bacteria such as *Gardnerella vaginalis* and *Mobiluncus mulieris* when colonized to the vaginal space, it leads to the development of bacterial vaginosis, preterm birth, and other sexually transmitted infections (STIs). A recent study indicates that bacterial EVs (bEVs) can be taken up by the vaginal epithelial cells and induce a Toll-like receptor 2 (TLR2)-dependent inflammatory response, leading to adverse reproductive outcomes (96). Another report delineates that vaginal symbiotic bacteria, *Lactobacillus* spp. release EVs, rich in unique proteins and metabolites, that protect CD4^+^ T-cells from HIV-1 infection probably by interfering with the viral attachment and entry into the target cells (97). Vaginal fibroblast-derived EVs of stress urinary incontinence (SUI) patients are shown to down-regulate the collagen content, proliferation, and migration of normal fibroblasts via the transfer of several differentially expressed proteins including tissue inhibitor of metalloproteinases 2 (TIMP-2), transforming growth factor-beta (TGFβ), and ATP-binding cassette sub-family C member 4 (ABCC4) (98). **Figure 6** depicts how EVs from different sources are associated with vaginal cancer.

#### EVs in uterine sarcoma

Very limited studies report the role of EVs in the progression of uterine sarcoma. A recent study demonstrates that the expression of miR-369-3p and miR-654-3p is significantly up-regulated in the EVs derived from the cell lines as well as the sera and tissues of uterine leiomyosarcoma (ULMS) patients which converts normal fibroblasts into CAFs, thereby contributing to the progression of uterine sarcoma (98).

#### EVs in vulval cancer

A single study demonstrates that EVs play a crucial role in the progression of vulval cancer. CAF-derived EVs are shown to confer cisplatin resistance to vulvar squamous cell carcinoma (VSCC) through the transfer of lncRNA urothelial cancer-associated 1 (UCA1) and regulating miR-103a/WEE1 axis (99).

**Figure 7:**
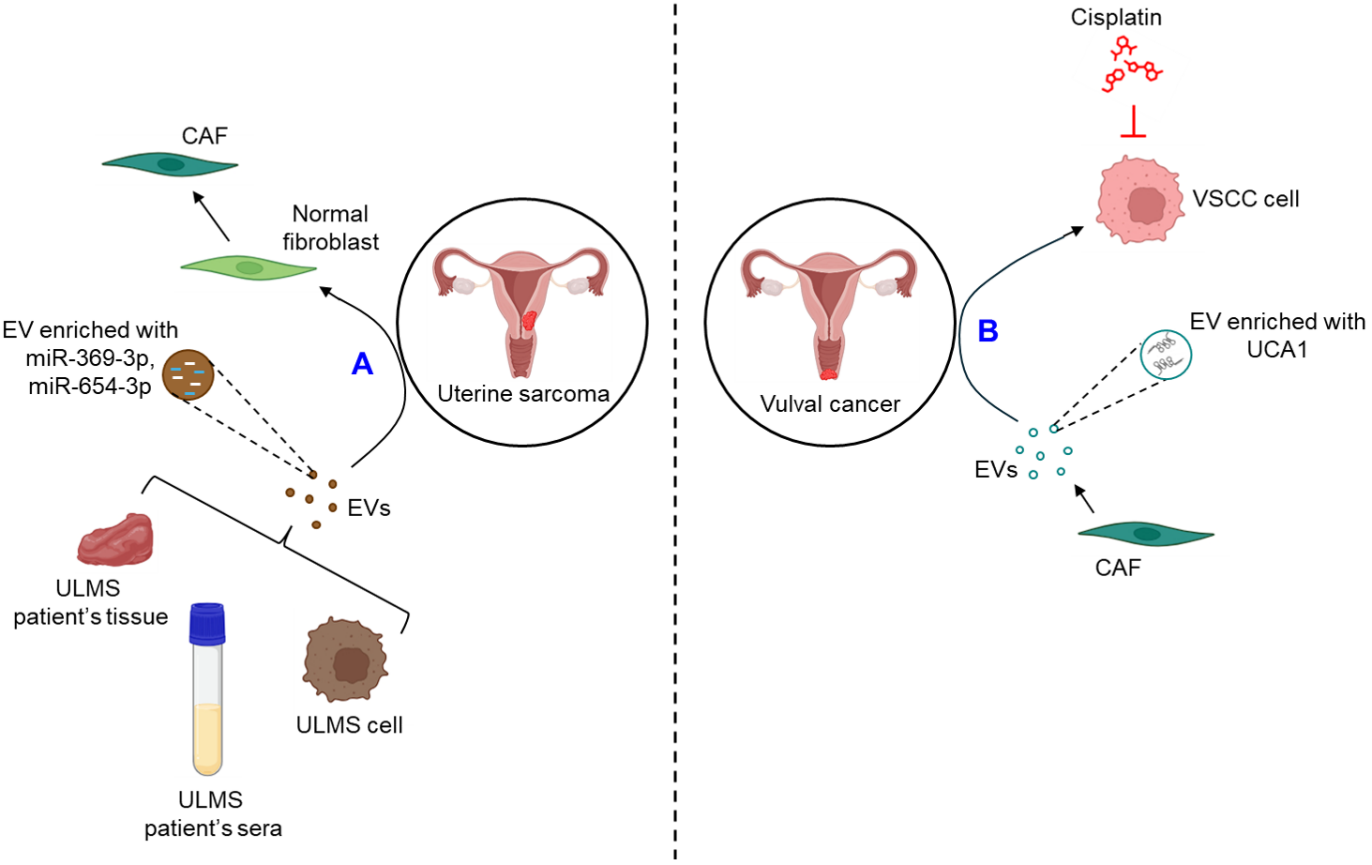
The role of EVs in uterine sarcoma and vulval cancer. **(A)** EVs released from ULMS cell lines or patient’s sera and tissues are enriched with miR-369-3p and miR-654-3p which convert normal fibroblasts into CAFs. **(B)** CAF-EVs are shown to be enriched with UCA1 which confers VSCC cells resistance against cisplatin. ULMS, uterine leiomyosarcoma; EVs, extracellular vesicles; miR, microRNA; CAF, cancer-associated fibroblast; UCA1, urothelial cancer-associated 1; VSCC, vulvar squamous cell carcinoma.

The role of EVs in the progression of GTD remains ill-defined. **Figure 7** briefly illustrates how EVs influence the progression of uterine sarcoma (**Figure 7A**) and vulval cancer (**Figure 7B**).

#### EVs as biomarkers for gynecological cancer

A biomarker is defined as a medical sign that indicates the medical state of a patient which can be accurately measured and is reproducible (105). The present section highlights a brief examples of how EVs serve as biomarkers for different types of gynecological cancers. **Table 3** also briefly delineates the role of EVs in different forms of gynecological cancers. For example, Zhou et al. have demonstrated that EVs from hypoxic cervical cancer cells are enriched with miR-152-3p which provides resistance against radiotherapy via targeting Kruppel-like factor 15 (KLF15) (106). As mentioned earlier, E6 oncoproteins of HPV 16/18 transfer Wnt7b mRNA from cervical cancer cells to endothelial cells through the EVs, thereby promoting endothelial proliferation and angiogenesis by β-catenin-dependent mechanisms, and thus can be considered as a potential biomarker for cervical cancer (77). Moreover, the expression of miR-125a-5p in the plasma EVs of cervical cancer patients is shown to be significantly lower as compared to the healthy individuals, thereby serving as a potential biomarker for cervical cancer diagnosis (107). A study by Ding et al. indicates that as compared to cervical intraepithelial neoplasia patients and normal controls, serum EVs of cervical cancer patients display a higher expression of lncRNA DLX6-AS1 which is positively correlated with lymph node metastasis, differentiation, shortened survival, and relapse (108). Therefore, lncRNA DLX6-AS1 in the serum EVs might serve as a promising marker for the prognosis and diagnosis of cervical cancer (108). Cervico-vaginal lavages of cervical cancer patients are shown to be enriched with EVs bearing signature lncRNAs, HOTAIR, MALAT1 and MEG3, making them early detection and diagnostic biomarkers for cervical cancer (109). By using ExoGAG, a highly efficient technology to enrich the EVs, Herrero et al. have demonstrated that endometrial cancer patients with high risk of recurrence exhibit higher expression of annexin A2 in the circulating EVs, thereby EVs’ annexin A2 level is considered as a prognostic biomarker for endometrial cancer (110). The expression of eight signature miRs, miR-383-5p, miR-10b-5p, miR-34c-3p, miR-449b-5p, miR-34c-5p, miR-200b-3p, miR-2110, and miR-34b-3p is shown to be dysregulated in the EVs isolated from pleural lavage of endometrial cancer patients as compared to EVs isolated from the ascitic fluid of control individuals, which marked them biomarkers for endometrial cancer (111). Moreover, miR-200c-3p expression in the urine EVs of endometrial cancer patients is significantly elevated as compared to EVs from patients without an established endometrial cancer which serve as a signature biomarker for endometrial cancer (112). Two signature circular RNAs, hsa_circ_0109046 and hsa_circ_0002577 are shown to be overexpressed in the serum EVs of endometrial cancer patients which is associated with the disease progression and considered as predictive biomarkers for endometrial cancer (113). Kuhlmann et al., by using next-generation sequencing (NGS)-based workflow, have identified miR-181a, miR-1908, miR-21, miR-486 and miR-223 to be over-expressed in the plasma EVs of platinum-resistant ovarian cancer patients, and thus designated these signature miRNAs as a promising biomarker for platinum-resistant ovarian cancer (114). In a cohort study, Lai et al. have demonstrated that three unique proteins, fibrinogen gamma gene (FGG), mucin 16 (MUC16), and apolipoprotein A4 (APOA4) are differentially expressed in the circulating EVs of ovarian cancer patients which can be used to screen patients with ovarian cancer (115). The complement C1r/C1s, Uegf, Bmp1 (CUB) domain-containing protein 1-positive (CDCP1+) EVs are shown to be significantly elevated in the ascites of ovarian cancer patients as compared to the benign counterparts, thereby CDCP1+ EVs is used as a biomarker for early response in ovarian cancer (116). Furthermore, epithelial ovarian cancer-released EVs are shown to transfer lncRNA, MALAT1 to endothelial cells, leading to angiogenesis, and thus considered as predictive biomarker for epithelial ovarian cancer (117). The expression of a circular RNA, circRNA051239 is significantly upregulated in the plasma EVs of epithelial ovarian cancer which targets miR-509-5p, leading to the induction of serine protease 3 (PRSS3), and promotion of cell proliferation and metastasis (118). Thus, EV-circRNA051239 can be considered as a diagnostic biomarker for metastatic epithelial ovarian cancer. As previously mentioned, EVs from cell lines, tissues, and sera of ULMS patients are enriched with miR-369-3p and miR-654-3p which transform normal fibroblasts into CAFs and hence EVs’ miR-369-3p and miR-654-3p can be used as prognostic and diagnostic biomarkers for ULMS (98).

**Table 3:** The role of EVs as biomarkers for different types of gynecological cancers.

Gynecological cancer type	EV component	Mechanism	Reference/s
Cervical cancer	miR-152-3p	EVs from hypoxia-triggered cervical cancer cells carry miR-152-3p which imparts cervical cancer resistance against radiotherapy via targeting KLF15	(106)
	Wnt7b mRNA	E6 oncoproteins of HPV 16/18 transfer Wnt7b mRNA from cervical cancer cells to the endothelial cells, inducing proliferation and angiogenesis by β-catenin-dependent pathway	(77)
	miR-125a-5p	The expression of miR-125a-5p in the plasma EVs of cervical cancer patients are significantly lower as compared to healthy individuals, serving as a potential biomarker for cervical cancer	(107)
	lncRNA DLX6-AS1	Serum EVs of cervical cancer show an elevated expression of lncRNA DLX6-AS1 which is also associated with lymph node metastasis, differentiation, shortened survival, and relapse, hence, can be considered as a promising biomarker for cervical cancer	(108)
	lncRNA HOTAIR, MALAT1, MEG3	The expression of HOTAIR, MALAT1, and MEG3 in the EVs derived from cervico-vaginal lavages of cervical cancer patients, are significantly upregulated, thereby considered as diagnostic biomarker for cervical cancer	(109)
Endometrial cancer	annexin A2	Annexin A2 in the circulating EVs of endometrial cancer patients is highly expressed which has the potential to be a prognostic biomarker for endometrial cancer	(110)
	miR-383-5p, miR-10b-5p, miR-34c-3p, miR-449b-5p, miR-34c-5p, miR-200b-3p, miR-2110, miR-34b-3p	The expression of eight signature miRs in the pleural lavage EVs of endometrial cancer is shown to be dysregulated in endometrial cancer as compared to EVs isolated from the ascitic fluid of control individuals thereby considered as biomarkers for endometrial cancer	(111)
	miR-200c-3p	miR-200c-3p expression in the urine EVs of endometrial cancer patients is shown to be well-elevated as compared to urine EVs of patients without an established endometrial cancer, thus serving as a biomarker	(112)
	hsa_circ_0109046, hsa_circ_0002577	Serum EVs of endometrial cancer patients are enriched with hsa_circ_0109046 and hsa_circ_0002577 which are considered as predictive biomarkers for endometrial cancer	(113)
Ovarian cancer	miR-181a, miR-1908, miR-21, miR-486, , miR-223	These five signature miRNAs are enriched in the plasma EVs of platinum-resistant ovarian cancer patients as compared to platinum- sensitive individuals and thus can be considered as promising biomarkers for platinum-resistant ovarian cancer	(114)
	FGG, MUC16, APOA4	Circulating EVs of ovarian cancer patients have differential expression of FGG, MUC16, and APOA4 which can be used to screen patients with ovarian cancer	(115)
	CDCP1	As compared to the benign ascites, ascites of ovarian cancer patients show an elevated level of CDCP1+ EVs which is used as a biomarker of early response in ovarian cancer	(116)
	lncRNA MALAT1	Epithelial ovarian cancer-derived EVs are shown to transfer MALAT1 to endothelial cells, thereby promoting angiogenesis, and thus considered as predictive biomarker for epithelial ovarian cancer	(117)
	circRNA051239	Plasma EVs of epithelial ovarian cancer show an enhanced expression of circRNA051239 which promotes proliferation and metastasis of epithelial cancer by targeting miR-509-5p and thus, inducing PRSS3 expression	(118)
Uterine sarcoma	miR-369-3p miR-654-3p	EVs from ULMS cells, tissues, and sera are enriched with miR-369-3p and miR-654-3p which transform normal fibroblasts into CAFs and thus, can be used as prognostic and diagnostic biomarkers for ULMS	(98)

KLF15, Kruppel-like factor 15; Wnt, wingless/integrated; lncRNA DLX6-AS1, long non-coding RNA distal-less homeobox 6 antisense RNA 1; EVs, extracellular vesicles; miR, microRNA; FGG, fibrinogen gamma gene; MUC16, mucin 16; APOA4, apolipoprotein A4; CDCP1, complement C1r/C1s, Uegf, Bmp1 (CUB) domain-containing protein 1; CAFs, cancer-associated fibroblasts; PRSS3, serine protease 3; ULMS, uterine leiomyosarcoma.

#### EVs in gynecological cancer diagnosis and prognosis

Early diagnosis and late follow-up become indispensable in the treatment of different types of cancer including gynecological cancer. In this regard, liquid biopsy of patients plays a significant role in the early diagnosis and prognosis of gynecological cancer. Emerging evidence has indicated that EVs released from the tumor cells often reside in body fluids like blood, urine, synovial fluid, saliva, breast milk etc. Therefore, analysis of EVs in the liquid biopsy of gynecological cancer patients often aids in the early diagnosis and prognosis of such cancer. In numerous occasions, it has been found that EVs themselves or their cargoes play a critical role in the diagnosis and prognosis of different types of gynecological tumors. A few examples are provided in the present section. For example, an increased expression of miR-21 and miR-146a is observed in the EVs collected from the cervicovaginal lavage fluid of cervical cancer patients as compared to normal subjects, hence these EV miRNAs can be used for diagnosing the cervical cancer (119). In another study, Zhang et al. have shown that three lncRNAs, Hox transcript antisense intergenic RNA (HOTAIR), maternally expressed gene 3 (MEG3), and MALAT1 (metastasis associated lung adenocarcinoma transcript 1) are enriched in the EVs derived from cervicovaginal lavages of cervical cancer patients which can be used for the detection and diagnosis of cervical cancer (109). Moreover, three signature miRNAs, miR-146a-5p, miR-151a-3p, and miR-2110 are over-expressed in the plasma EVs of cervical cancer patients and can be served as diagnostic and prognostic biomarkers for cervical cancer (120). A study by Herrero et al. has demonstrated that the expression of annexin A2 is significantly higher in the plasma EVs of endometrial cancer patients which is associated with disease recurrence and thus can be considered as a diagnostic and prognostic biomarker for endometrial cancer (110). In addition, miR-15a-5p, miR-106b-5p, and miR-107 are significantly enriched in the plasma EVs of endometrial cancer which is correlated with the disease progression, thus are considered as promising biomarkers for early diagnosis of endometrial cancer (121). An increased expression of miR-95 and decreased expression of miR-205 in the serum EVs of endometrial cancer renders them promising prognostic biomarkers for endometrial cancer (122). In ovarian cancer patients, a down-regulation of miR-1260a, miR-7977, and miR-192-5p expression is observed in the plasma EVs with the potential of considering as diagnostic and prognostic biomarkers for ovarian cancer (123). The expression of hepatocyte growth factor (HGF), signal transducer and activator of transcription 3 (STAT3), and interleukin-6 (IL-6) is shown to be significantly higher in the serum EVs of early stage high grade ovarian cancer as compared to benign and late stage tumor which can be used for early diagnosis of ovarian cancer (124). Moreover, claudin-4 is incorporated into the EVs derived from ovarian cancer cells which is retained in the peripheral blood, thereby likely to be used as a prognostic marker for ovarian cancer (125). In case of ULMS, the expression of miR-654-3p and miR-369-3p in the serum EVs is shown to be significantly higher as compared to myoma patients which can be used in the diagnosis and prognosis of ULMS (126). **Table 4** briefly illustrates how EVs contribute to the early diagnosis and prognosis of various gynecological cancers.

**Table 4:** The role of EVs in the diagnosis of different types of gynecological cancers.

Gynecological cancer type	EV origin	EV component	Reference/s
Cervical cancer	Cervicovaginal lavage fluid	miR-21, miR-146a 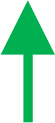	(119)
	Cervicovaginal lavage fluid	HOTAIR, MEG3, MALAT1 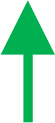	(109)
	Plasma	miR-146a-5p, miR-151a-3p, miR-2110 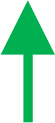	(120)
Endometrial cancer	Plasma	Annexin A2 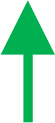	(110)
	Plasma	miR-15a-5p, miR- 106b-5p, miR-107 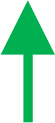	(121)
	Serum	miR-95 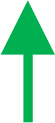 miR-205 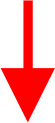	(122)
Ovarian cancer	Plasma	miR-1260a, miR- 7977, miR-192-5p 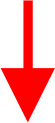	(123)
	Serum	HGF, STAT3, IL-6 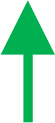	(124)
	Peripheral blood	Claudin-4 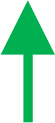	(125)
ULMS	Serum	miR-654-3p, miR-369-3p 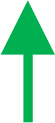	(126)

miR, microRNA; HOTAIR, Hox transcript antisense intergenic RNA; MEG3, maternally expressed gene 3; MALAT1, metastasis associated lung adenocarcinoma transcript 1; HGF, hepatocyte growth factor; STAT3, signal transducer and activator of transcription 3; IL-6, interleukin 6. (
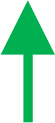
) indicates up-regulation; (
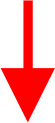
) indicates down-regulation

## Conclusion and future direction

Gynecological cancer is considered as one of the major causes of cancer-related death among women worldwide. The treatment measures for gynecological cancer include surgery, radiation oncology, and medical oncology. However, despite advancement in the treatment measures, gynecological cancers continue to be the leading cause of morbidity and mortality of patients. The major reasons behind the increased mortality of gynecological cancers include the detection of tumors in the later stages, limited treatment options, and disease recurrence. Emerging evidence indicates that EVs play an important role in the progression of gynecological cancer by various mechanisms. The present review highlights how tumor cells communicate with other cells in the tumor microenvironment and vice versa through the release of EVs, thereby aiding in the progression of cancer. It has been shown in multiple occasions that EVs’ cargo plays an important role in the progression of gynecological cancer via modulating the phenotype of the EVs-fused recipient cells, and thus considered as prognostic and diagnostic biomarkers for gynecological cancer. Therefore, targeting EVs biogenesis, EVs’ cargo, and the uptake of EVs offer promising therapeutic strategies in restricting the progression of gynecological cancers. On top of this, due to easy uptake mechanism of the EVs, bioengineered EVs often show promising results in the treatment of different cancer types including gynecological cancer. Moreover, mesenchymal stem cell-derived EVs show anticancer properties in the context of gynecological tumors. Therefore, EVs can be used as a promising therapeutic machinery in gynecological cancer depending on the cellular origin. Moreover, EVs readily fuse with the recipient cells and are capable of avoiding host immune response. These trigger the use of EVs as a vehicle in which a therapeutic drug can be entrapped and efficiently transferred to the target recipient cells. In this regard, EV-mediated drug delivery can be an effective therapeutic approach in the treatment of various forms of gynecological tumors. However, a better understanding of EVs biogenesis, functions, and heterogeneity will facilitate the development of advanced EV-based therapeutic strategies against gynecological cancer.
